# Diagnostic Methods Used to Classify Confirmed and Probable Cases of Spotted Fever Rickettsioses — United States, 2010–2015

**DOI:** 10.15585/mmwr.mm6810a3

**Published:** 2019-03-15

**Authors:** Alison M. Binder, Kristen Nichols Heitman, Naomi A. Drexler

**Affiliations:** 1 Division of Vector-Borne Diseases, National Center for Emerging and Zoonotic Infectious Diseases, CDC.

Spotted fever rickettsioses (SFR), including Rocky Mountain spotted fever (RMSF), are nationally notifiable diseases in the United States caused by spotted fever group *Rickettsia*. The annual incidence of SFR increased from 1.7 cases per 1 million persons in 2000 to 13.2 in 2016 (*1*,*2*). Although this demonstrates a substantial increase in SFR cases, the actual magnitude of the increase is questionable because the current case definition allows for nonspecific laboratory criteria to support diagnosis (*3*). To analyze the quality of laboratory data used to support the diagnosis of SFR cases with illness onset during 2010–2015, CDC examined supplementary case report forms. Among 16,807 reported cases, only 167 (1.0%) met the confirmed case definition, and the remaining 16,640 (99.0%) met the probable case definition. The most common supportive laboratory evidence for probable cases was elevated immunoglobulin G (IgG) antibody titer by indirect immunofluorescence assay (IFA), which was reported for 14,784 (88.8%) probable cases. Antibodies to spotted fever group *Rickettsia* can persist for months or years following infection, making a single antibody titer unreliable for diagnosing incident disease without a convalescent specimen. Increased use of molecular assays and use of paired and appropriately timed IFA IgG testing practices could improve understanding of SFR epidemiology and increase the accuracy of disease incidence estimates.

SFR are bacterial diseases spread by the bite of infected ticks. SFR are difficult to diagnose because early signs and symptoms are nonspecific and acute-phase diagnostic tests are not widely available. SFR are typically described as acute febrile illnesses with headache, malaise, rash, and, in some cases, eschars. SFR cause mild to severe illness depending on the causative agent. For example, *Rickettsia parkeri* rickettsiosis is typically milder, whereas RMSF, caused by *Rickettsia rickettsii,* the most severe tickborne disease in the United States, can cause severe illness and death (estimated case fatality rate = 5%–10%) (*4*). Doxycycline is the treatment of choice for all patients with SFR; delay in treatment is associated with an increased risk of death (*4*). There is growing awareness that an increasing percentage of SFR are not cases of RMSF, but represent disease caused by similar, less-pathogenic *Rickettsia* species (*5*). However, spotted fever group *Rickettsia* antigens cross-react, and routine serologic assays cannot provide conclusive species-specific diagnoses (*6*).

CDC is notified of SFR cases through two passive surveillance systems, the National Notifiable Diseases Surveillance System (NNDSS) and Tickborne Rickettsial Disease case report forms. Supplemental data reported through case report forms describe clinical course and diagnostic testing. Tickborne Rickettsial Disease case report forms submitted to CDC by May 1, 2018, for cases with illness onset during 2010–2015 were included in this analysis. SFR cases were identified using the Council of State and Territorial Epidemiologist (CSTE) case criteria (*3*). CSTE laboratory criteria for confirmed SFR includes seroconversion (defined as a fourfold change in anti-SFR IgG antibody titers) by IFA (using paired serum specimens, one taken in the first week of illness and a second taken 2–4 weeks later) or polymerase chain reaction (PCR), immunohistochemistry (IHC), or culture. Laboratory criteria for probable SFR includes serologic detection of anti-SFR IgG or immunoglobulin M (IgM) antibodies by a number of methods, including IFA, enzyme immunoassay/enzyme-linked immunosorbent assay (EIA/ELISA), dot-ELISA, or latex agglutination. IgG or IgM values of ≥1:64 by IFA were considered positive. All analyses were performed using SAS software (version 9.4, SAS Institute).

During 2010–2015, CDC received 16,807 case reports of SFR meeting the probable or confirmed case definition. The number of cases reported annually increased from 1,617 in 2010 to 2,275 in 2015. As the number of annual cases increased, the percentage of confirmed cases decreased from 1.9% in 2010 to 0.7% in 2015. Overall, SFR was confirmed in 167 (1.0%) reported cases, including 102 by seroconversion; 66 by PCR, IHC, or culture; and one by both seroconversion and PCR (Figure). Among confirmed cases, the median interval from illness onset to first specimen collection was 4 days (interquartile range [IQR] = 1–6 days) (Table 1), and IFA IgG testing was reported for 124 (74.3%) first specimens, 91 (73.4%) of which were positive, including 46 with titers ≥1:128. Among the 112 confirmed cases with at least two specimens reported, the median interval from first to second specimen collection was 19 days (IQR = 16–23); 107 (95.5%) second specimens were tested for IgG by IFA, 104 (97.2%) of which were positive.

**FIGURE:**
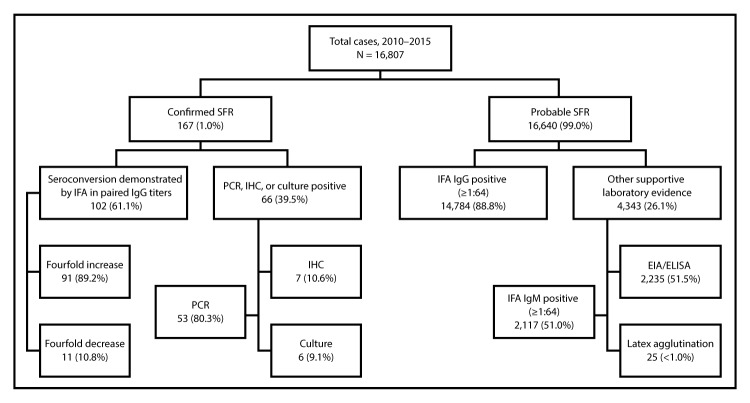
Summary of laboratory methods used to classify confirmed and probable cases of spotted fever rickettsiosis (SFR) — United States, 2010–2015 *^,†,§^ **Abbreviations:** EIA/ELISA = enzyme immunoassay/enzyme-linked immunosorbent assay; IFA = immunofluorescence assay; IgG = immunoglobulin G; IgM = immunoglobulin M; IHC = immunohistochemistry; PCR = polymerase chain reaction. * “Confirmed SFR” and “Probable SFR” classifications are mutually exclusive; cases cannot be included in both categories. ^†^ Percentages for “Seroconversion demonstrated by IFA in paired IgG titers” and “PCR, IHC, or culture positive” might not sum to 100% because categories are not mutually exclusive. Percentages for “IFA IgG positive” and “Other supportive laboratory evidence” also might not sum to 100% because categories are not mutually exclusive. ^§^ One case was reported confirmed by both “PCR” and “Seroconversion demonstrated by IFA in paired IgG titers.”

**TABLE 1:** Laboratory characteristics of confirmed and probable spotted fever rickettsiosis cases (SFR) — United States, 2010–2015

Characteristic	Confirmed* (n = 167)	Probable^†^ (n = 16,640)
	No. (%)	No. (%)
**First specimen collection and test, all cases (N = 16,807)**		
**Interval from symptom onset to first specimen collection (days)**		
0–7	129 (77.2)	8,515 (51.2)
≥8	20 (12.0)	4,375 (26.3)
Unknown/Not reported	18 (10.8)	3,750 (22.5)
**Median (IQR)**	4 (1–6)	5 (2–11)
**Test characteristics**		
IFA IgG performed	124 (74.3)	14,911 (89.6)
**IFA IgG titer distribution (% among those tested)^§^**		
<1:64	33 (26.6)	337 (2.3)
≥1:64	91 (73.4)	14,574 (97.7)
≥1:128	46 (37.1)	7,056 (47.3)
**Second specimen collection and test, cases with at least two specimens (n = 3,054)**		
No. (%) of second specimens	112 (67)	2,942 (17.7)
**Interval from first to second specimen collection (days)**		
0–13	4 (3.6)	486 (16.5)
14–28	104 (92.9)	520 (17.7)
≥29	3 (2.7)	782 (26.6)
Unknown/Not reported	1 (0.9)	1,154 (39.2)
**Median (IQR)**	19 (16–23)	24 (13–47)
**Test characteristics**		
IFA IgG performed	107 (95.5)	1,618 (55.0)
**IFA IgG titer distribution^§^**		
<1:64	3 (2.8)	67 (4.1)
≥1:64	104 (97.2)	1,549 (95.7)
≥1:128	92 (90.0)	957 (59.1)

**Abbreviations:** IFA = indirect immunofluorescence assay; IgG = immunoglobulin G; IQR = interquartile range.

* Laboratory-confirmed criteria: serologic evidence of a fourfold change in IgG-specific antibody titer reactive with spotted fever group antigen by indirect IFA between paired serum specimens (one taken in the first week of illness and a second 2–4 weeks later), or by polymerase chain reaction, immunohistochemistry, or cell culture. A confirmed SFR case is clinically compatible if it meets clinical evidence criteria (i.e., any reported fever and one or more of the following: rash, eschar, headache, myalgia, anemia, thrombocytopenia, or any hepatic transaminase elevation) and is laboratory-confirmed.

^†^ Laboratory-supportive criteria: serologic evidence of elevated IgG or immunoglobulin M antibody reactive with spotted fever group antigen by IFA, ELISA, dot-ELISA, or latex agglutination. A probable SFR case is clinically compatible and has supportive laboratory results.

^§^ IFA IgG titer results are considered positive if ≥1:64.

Overall, 16,640 (99.0%) cases met criteria for probable SFR. Elevated IFA IgG titers in at least one specimen was the most commonly reported supportive laboratory finding (14,784 cases, 88.8%); (Figure). Elevated IFA IgM titers were reported for 2,117 (12.7%) probable cases, positive ELISA results were reported for 2,235 (13.4%), and positive latex agglutination was reported for 25 (<1.0%). Use of dot-ELISA was not reported. Among probable cases, the median interval from illness onset to first specimen collection was 5 days (IQR = 2–11 days) (Table 1); 77.2% of specimens were collected within the first week of illness. Among all 16,640 probable cases, IFA IgG testing was performed on the first specimen for 14,911 (90%). Collection of a second specimen was reported for 2,942 (19.7%) of all probable cases, 1,618 (55.0%) of which were tested by IFA IgG. Overall, paired specimen testing by IFA IgG within recommended date ranges was reported infrequently among probable cases (218 cases, 1.3%) (Table 2). Most probable cases were supported by a single elevated IFA IgG titer (13,557 cases, 81.5%).

**TABLE 2:** Reasons for failure to meet confirmation criteria* among probable^†^ spotted fever rickettsiosis cases (N = 16,640) — United States, 2010–2015

Reason	No. (%)
Paired IFA IgG testing performed within recommended date range, without evidence of seroconversion	218 (1.3)
Paired IFA IgG testing performed outside of recommended date range	1,268 (7.6)
Supportive evidence demonstrated with IFA IgM, ELISA, dot-ELISA, or latex agglutination only	1,597 (9.6)
Single positive IFA IgG titer^§^	13,557 (81.5)

**Abbreviations:** ELISA = enzyme-linked immunosorbent assay; IFA = indirect immunofluorescence assay; IgG = immunoglobulin G; IgM = immunoglobulin M; IQR = interquartile range.

* Laboratory confirmed criteria: serological evidence of a fourfold change in IgG-specific antibody titer reactive with spotted fever group antigen by indirect IFA between paired serum specimens (one taken in the first week of illness and a second 2–4 weeks later), or by polymerase chain reaction, immunohistochemistry, or cell culture. A confirmed SFR case is clinically compatible (meets clinical evidence criteria: any reported fever and one or more of the following: rash, eschar, headache, myalgia, anemia, thrombocytopenia, or any hepatic transaminase elevation) and is laboratory-confirmed.

^†^ Laboratory-supportive criteria: serologic evidence of elevated IgG or immunoglobulin M antibody reactive with spotted fever group antigen by IFA, enzyme-linked immunosorbent assay (ELISA), dot-ELISA, or latex agglutination. A probable SFR case is clinically compatible and has supportive laboratory results.

^§^ IFA IgG titer results are considered positive if ≥1:64.

## Discussion

The goal of SFR surveillance is to provide information to health care providers and public health officials about the temporal, geographic, and demographic occurrence of SFR and to facilitate prevention and control (*3*). During 2010–2015, only 1.0% of SFR cases reported to CDC via case report forms met the criteria for a confirmed SFR case. The majority of probable cases were not confirmed because of incomplete serologic testing. In addition, PCR, IHC, or culture were infrequently used for case confirmation, despite the high specificity of these techniques.

IgM antibodies, latex agglutination, and ELISA testing provide insufficient evidence to confirm a new SFR illness; use of such tests hinders full understanding of SFR epidemiology and the incidence of disease in the United States (*7*). IgG antibodies against spotted fever group *Rickettsia* can remain elevated for months or years following exposure and subsequent clinical recovery from illness. National studies have estimated up to 6% SFR seropositivity in the U.S. population (*8*). Other localized seroprevalence studies in areas with endemic SFR have found rates as high as 22% (*9*). Therefore, it is impossible to differentiate a single elevated IgG titer associated with acute illness from previous infection given the high background seroprevalence of these infections. Because of this, single antibody titers, even when collected during the course of an illness clinically compatible with SFR, are not reliable for diagnosing an incident infection. Health care providers and public health practitioners should be aware of the limited interpretability of unpaired tests and encourage patients to return for convalescent serologic testing. Species-specific real-time PCR assays are now available at some qualified state and local public health laboratories; increased use of these assays will be important for accurately characterizing infections with SFR and identifying the etiologic agent (*10*).

The findings in this report are subject to at least three limitations. First, SFR surveillance is a passive system, and data might be biased by differences in case investigation thresholds and nonrandom reporting. The quality of passive surveillance data depends on clinician awareness and use of appropriate diagnostic tests, documentation of epidemiologic factors, and timely reporting to public health officials. As such, cases described in this report might not be generalizable to all SFR cases. Second, this analysis only included cases reported using case report forms and might not be representative of all cases reported to NNDSS. Finally, supplemental SFR surveillance collects limited clinical information, restricting the ability to evaluate trends and disease severity associated with species-specific diagnoses.

This analysis highlights the importance of collecting appropriately timed specimens for serologic confirmation and use of molecular diagnostic tests. Because of the reliance on serologic methods, the causative agent is seldom identified. Molecular methods are not widely available for commercial use and are rarely used to confirm SFR. Beginning in 2018, real-time molecular assays have been made available to qualified state and local laboratories through CDC’s Laboratory Response Network. In addition to increased use of molecular detection, eliminating diagnostic tests of limited interpretability as supportive evidence from the case definition of SFR surveillance could be important for understanding trends in species-specific SFR cases in the United States.

**Summary**

**What is already known about this topic?**

Spotted fever rickettsioses (SFR) are nationally notifiable diseases caused by spotted fever group *Rickettsia*. SFR incidence has steadily increased since 2000; however, the majority of cases fail to meet criteria for confirmation.

**What is added by this report?**

A total of 16,807 SFR supplemental case report forms were provided to CDC with illness onset during 2010–2015; 1.0% met criteria for confirmation. Reasons for nonconfirmation included failure to submit a second serum specimen and low use of molecular diagnostic techniques.

**What are the implications for public health practice?**

Increased use of molecular assays, collecting appropriately timed serum specimens, and elimination of unreliable laboratory criteria could be important for understanding trends in SFR epidemiology in the United States.
